# *MiR-629-5p* promotes colorectal cancer progression through targetting CXXC finger protein 4

**DOI:** 10.1042/BSR20180613

**Published:** 2018-08-29

**Authors:** Jinlai Lu, Shuirong Lu, Jingze Li, Qi Yu, Lang Liu, Qin Li

**Affiliations:** Department of Gastroenterology, Shanghai East Hospital, Shanghai Tongji University, Shanghai 200123, P.R. China

**Keywords:** colorectal cancer, CXXC4, miR-629-5p, progression

## Abstract

*MiR-629-5p* has been shown to function as a tumor promoter in some types of cancer. However, the role of *miR-629-5p* in colorectal cancer remains unclear. Here, the significant up-regulation of *miR-629-5p* in colorectal cancer tissues and cell lines was observed. Overexpression of *miR-629-5p* showed a positive effect on cell proliferation and migration. The enhanced *miR-629-5p* level also suppressed cell apoptosis and resulted in a low Bax level and a high Bcl-2 level. Further down-regulating *miR-629-5p* demonstrated opposite effects. CXXC finger protein 4 (CXXC4) was predicted as a direct target of *miR-629-5p*. Dual-luciferase reporter and Western blotting assays exhibited *miR-629-5p* directly bound to the 3′UTR of CXXC4 and then down-regulated its expression at post-transcriptional level. CXXC4 knockdown rescued the decreased cell proliferation and migration and the enhanced cell apoptosis induced by inhibiting *miR-629-5p* expression. Notably, overexpression of *miR-629-5p* also conferred 5-fluorouracil sensitivity, which was partly abrogated by coexpression of CXXC4. Overall, the results presented here suggest that *miR-629-5p* functions as a tumor promoter by improving proliferation and migration and repressing apoptosis and 5-FU sensitivity in colorectal cancer progression by directly down-regulating CXXC4.

## Introduction

Colorectal cancer is a frequent digestive malignancy, causing more than 600 thousand deaths annually worldwide [1]. Although surgical resection and some combined therapies have been clinically applied, therapeutic effects on advanced colorectal cancer remain poor. Failure in treating advanced colorectal cancer is a main cause of colorectal cancer related deaths. Therefore, early diagnosis is critical to improve the prognosis and to reduce the mortality rate. However, more than 60% colorectal cancer cases are diagnosed at advanced stage due to the asymptomatic nature of early stage colorectal cancer [2]. Thus, it is still imperative to develop effective biomarkers and clinical strategies for curing colorectal cancer.

MiRNAs are small non-coding RNAs that post-transcriptionally repress the translation or promote the degradation of targetted mRNAs [3]. Generally, one single miRNA is involved in the regulation of multiple target genes via binding to their 3′UTR [4]. Therefore, miRNAs are important regulators in biological process, including cell proliferation, migration, and apoptosis [5,6]. It has been proved that the aberrant expression of miRNAs is a common feature of human malignancy [7]. Emerging evidence have revealed that miRNAs can be oncogenes or tumor suppressors via regulating cell progression [8,9]. Previous studies showed that *miR-629-5p* was responsible for the increased risk of lung cancer [10]. *MiR-629-5p* also promoted cell motility and invasion in renal cell carcinoma by regulating TGFβ/Smad signaling [11]. In ovarian cancer, *miR-629-5p* promoted cell proliferation, migration, and invasion by directly repressing testis-specific Y-like protein 5. Additionally, *miR-629-5p* also acted as an oncogene in human pancreatic cancer and cervical cancer cell lines [12,13]. However, the biological function of *miR-629-5p* in colorectal cancer is still ambiguous.

CXXC finger protein 4 (CXXC4) is a novel tumor suppressor. In renal cell carcinoma, lower CXXC4 level was associated with promoted malignant phenotype [14]. In gastric cancer, the down-regulated CXXC4 contributed to proliferation [15,16] and anti-apoptosis of cells [17]. Besides, promoter methylation and aberrant expression of CXXC4 were also observed in head and neck squamous cells [18]. Former studies proved that some proteins regulated the expression of CXXC4 [16,19], but the potential regulation of CXXC4 by miRNA is still unclear. In this research, we tried to explore the functions of *miR-629-5p* and CXXC4 in colorectal cancer and to reveal the potential relationship between these molecules.

## Materials and methods

### Clinical samples and cell culture

The written informed consent from all enrolled patients was obtained before operation. All the studies were approved by the Ethical Committee of Shanghai Tongji University. The colorectal cancer tissues and the adjacent normal tissues were obtained form 30 patients undergoing surgery in Shanghai East hospital from 2014 to 2016. None of the patients had received chemo, radiation, or immune therapy before the operation. The collected specimens were snap-frozen and then preserved in liquid nitrogen.

Human colorectal cancer cell lines SW480 (catalog number: 3131C0001000700172, primary tumor-derived), LoVo (catalog number: 3131C0001000700082, distal metastatic), HT29 (catalog number: 3131C0001000700103, primary tumor-derived), HCT116 (catalog number: 3131C0001000700099, primary tumor-derived), and SW620 (catalog number: 3131C0001000700101, lymph node metastatic derivatives of SW480) were purchased form the Type Culture Collection of the Chinese Academy of Sciences (Shanghai, China). The normal colon epithelial cell line NCM460 (catalog number: BNF-3068) was purchased from Shanghai Rongbai Biological Technology Co., Ltd. (Shanghai, China). Cell lines were cultivated in RPMI-1640 medium (Gibco; Thermo Fisher Scientific, Inc., Waltham, MA, U.S.A.) supplemented with 10% FBS (Thermo Fisher Scientific), streptomycin (100 U/ml) (Thermo Fisher Scientific), and penicillin (100 U/ml) (Thermo Fisher Scientific) at 37°C under 5% CO_2_.

### Quantitative real-time PCR

Total RNA from tissues or cancer cells was isolated by TRIzol reagent (Invitrogen, CA, U.S.A.) and then used for cDNA synthesis according to the manual of PrimeScript™ RT Reagent Kit with gDNA Eraser (Takara Biotechnology Co., Ltd., Dalian, China). Further quantitative real-time PCR (qRT-PCR) assay of target genes was carried out using SYBR® Advantage® qPCR Premix (Takara). The expression level of miRNA was determined by Mir-X™ miRNA First Strand Synthesis Kit (Takara) and Mir-X™ miRNA qRT-PCR SYBR® Kit (Takara) as described by manuals. The qRT-PCR assay was performed by a StepOnePlus™ Real-Time PCR Systems (Thermo Fisher Scientific). GAPDH and U6 were applied as internal controls for analyzing the mRNA levels of proteins and *miR-629-5p*, respectively. The transcriptional level was analyzed with the 2^−ΔΔ*C*^_t_ analytical method. Experiments were performed three times and each sample was analyzed in triplicate. Primers used here were listed in Table 1.

**Table 1:** Primers used in the present study

Name	Sequence (5′–3′)
CXXC4 F	CTCATCAACTGTGGCGTCTG
CXXC4 R	TTAGTTTGCCCTTCATTTCC
U6 F	CTCGCTTCGGCAGCACA
U6 R	AACGCTTCACGAATTTGCGT
GAPDH F	GGAGTCAACGGATTTGGT
GAPDH R	GTGATGGGATTTCCATTGAT
pcDNA-CXXC4 F	CCCAAGCTTCTCGAGATGCACCACCGAAACGACTCC
pcDNA-CXXC4 R	CCGGAATTCTTAAAAGAACCATCGGAATGCTTCA

### Genetic manipulation and transfection

*MiR-629-5p* mimic, *miR-629-5p* inhibitor, *miR-629-5p* mimic control (mimic Con), and *miR-629-5p* inhibitor control (inhibitor Con) were provided by Guangzhou RiboBio Co., Ltd. (Guangzhou, China). CXXC4-specific siRNA and a scrambled siRNA (si-Con) were also provided by RiboBio. The DNA encoding CXXC4 protein was cloned from cDNA of LoVo cell and then inserted into plasmid pcDNA3.1(+) for overexpressing CXXC4, generating the pcDNA-*CXXC4*. Gene sequencing was provided by Sangon Biotech (Shanghai) Co., Ltd. (Shanghai, China). Transfection was then performed at the cell concentration of 50–70% confluence using Lipofectamine 2000 reagent (Invitrogen) according to the manual. Primers used here were listed in Table 1.

### Cell viability

Cell were seeded in a 96-well plate at a density of 800 cells/well for 0, 24, 48, and 72 h. After cultivation, cell viability was determined by a CCK8 assay kit (Beyotime, Shanghai, China) at 450 nm according to the manual. Experiments were performed three times and each sample was analyzed in triplicate.

### Transwell migration assay

Cell migration assay were performed in Transwells with 8-μm polycarbonate membrane pores (Corning, U.S.A.). After transfection for 24 h, cells were trypsinized and suspended in serum-free RPMI-1640 medium containing 10% BSA and then transferred in the upper chamber (5 × 10^4^ cells/well). The lower chamber was filled with RPMI-1640 medium supplemented with 10% FBS. After 48 h incubation, the migratory cells were fixed with methanol and then stained with Crystal Violet. Penetrated cells (five fields per chamber) were counted under an inverted microscope.

### Bioinformatics analysis and dual-luciferase reporter assay

The potential target genes of *miR-629-5p* were analyzed by online software TargetScan 7.1 (http://www.targetscan.org/vert_71/). The native or mutant 3′UTR of CXXC4 was amplified and cloned into plasmid pMIR-Reporter (Thermo Fisher), generating the plasmids pMIR-wt and pMIR-mut, respectively. The generated vectors were cotransfected with *miR-629-5p* mimic, *miR-629-5p* inhibitor, mimic Con, or inhibitor Con into LoVo cells. After 48 h, the luciferase activity was determined by the Dual-Luciferase Reporter Assay System (Promega, WI, U.S.A.). The relative firefly luciferase activity was presented by normalizing to *Renilla* luciferase activity.

### Western blotting

The treated cells were collected and washed twice with PBS buffer. Cells were then lysed by RIPA lysis buffer (Beyotime Biotechnology, Shanghai, China) for total protein extraction. The BCA protein assay kit (Beyotime Biotechnology) was applied for determining protein concentration. Protein with equivalent amounts were subsequently separated by SDS/PAGE and then transferred on to a PVDF membrane. After blocking, the membranes were incubated with primary antibodies against CXXC4 (ab105400, 1:500), BCL-2 (ab32124, 1:1000), Bax (ab32503, 1:1000), and β-actin (ab8227, 1:3000) (Abcam, Cambridge, U.K.) overnight at 4°C. After that, the membranes were incubated with secondary antibody (ab205718) (Abcam) and exposed using an Ultrasensitive ECL Chemiluminescence kit (Sangon Biotech) according to the manual.

### Cell apoptosis analysis

Cell apoptotic rate was then measured by an FITC labeled Annexin V Apoptosis Detection Kit (BD Biosciences, U.S.A.). Cells were collected, trypsinized, and then washed twice with cold PBS buffer. The obtained cells were resuspended in 300 μl binding buffer and then labeled with 5 μl Annexin V-FITC for 15 min in the dark. After that, cells were stained by 5 μl PI for 5 min and 200 μl binding buffer was added before analysis by flow cytometry.

### Statistical analysis

All data were analyzed by SPSS 17.0 (SPSS Inc., Chicago, U.S.A.). Data are presented as mean ± S.D. of at least three independent experiments. Comparison between two experimental groups was performed by Student’s *t*test. ANOVA was applied in the comparison of multiple groups. Samples with *P*-values of <0.05 were considered statistically different.

## Results

### Up-regulation of *miR-629-5p* in colorectal cancer tissues and cell lines

**Figure 1:**
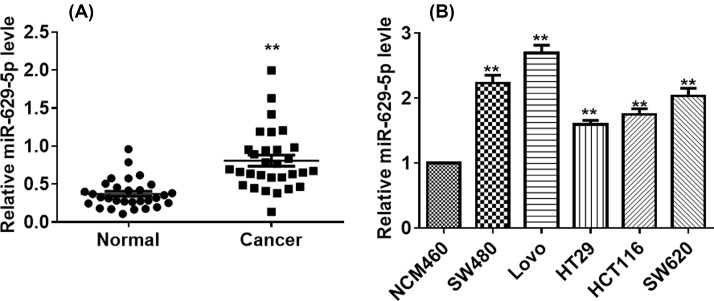
*miR-629-5p* was up-regulated in colorectal cancer tissues and cell lines (**A**) qRT-qPCR analysis of the expression level of *miR-629-5p* in colorectal cancer tissues (*n*=30) and normal tissues (*n*=30). U6 RNA was used as the internal control. **P<0.01, Student’s *t* test. Data were obtained from three technical replicates. (**B**) Relative expression of *miR-629-5p* in NCM460 and colorectal cell lines was determined by qRT-PCR. **P<0.01, one-way ANOVA test. Data were obtained from three technical replicates.

The mRNA levels of *miR-629-5p* were analyzed by qRT-PCR in the collected colorectal tumor tissues and the adjacent tumor-free tissues. The average expression level of *miR-629-5p* in tumor tissues was higher than that in the adjacent normal tissues (Figure 1A), indicating the up-regulation of *miR-629-5p* in colorectal cancer tissues. Expressions of *miR-629-5p* in colorectal cancer cell lines (SW480, LoVo, HT29, HCT116, and SW620) and a normal colorectal epithelium cell line (NCM460) were also examined. The expression levels of *miR-629-5p* in the tested cancer cell lines were all higher than that in the normal cell line (Figure 1B). In addition, the lowest *miR-629-5p* level was observed in HT29 cells. LoVo cells showed the highest expression level of *miR-629-5p* and then was selected for further study.

### Effect of *miR-629-5p* on cell proliferation and migration in LoVo cells

**Figure 2:**
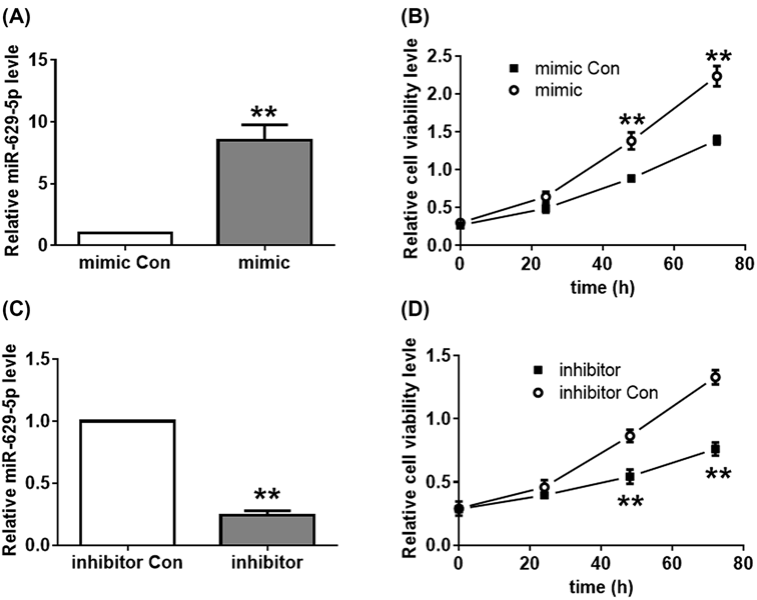
Effect of *miR-629-5p* on cell proliferation of LoVo cells (**A**) The level of *miR-629-5p* in LoVo cells transfected with *miR-629-5p* mimic. **P<0.01, Student’s *t* test. Data were obtained from three biological replicates. (**B**) Effect of the overexpressed *miR-629-5p* on the viability of LoVo cells was determined by the CCK-8 assay. **P<0.01, Student’s *t* test. Data were obtained from three biological replicates. (**C**) The level of *miR-629-5p* in LoVo cells transfected with *miR-629-5p* inhibitor. **P<0.01, Student’s *t* test. Data were obtained from three biological replicates. (**D**) Effect of the repressed *miR-629-5p* on the viability of LoVo cells was determined by the CCK-8 assay. **P<0.01, Student’s *t* test. Data were obtained from three biological replicates.

**Figure 3:**
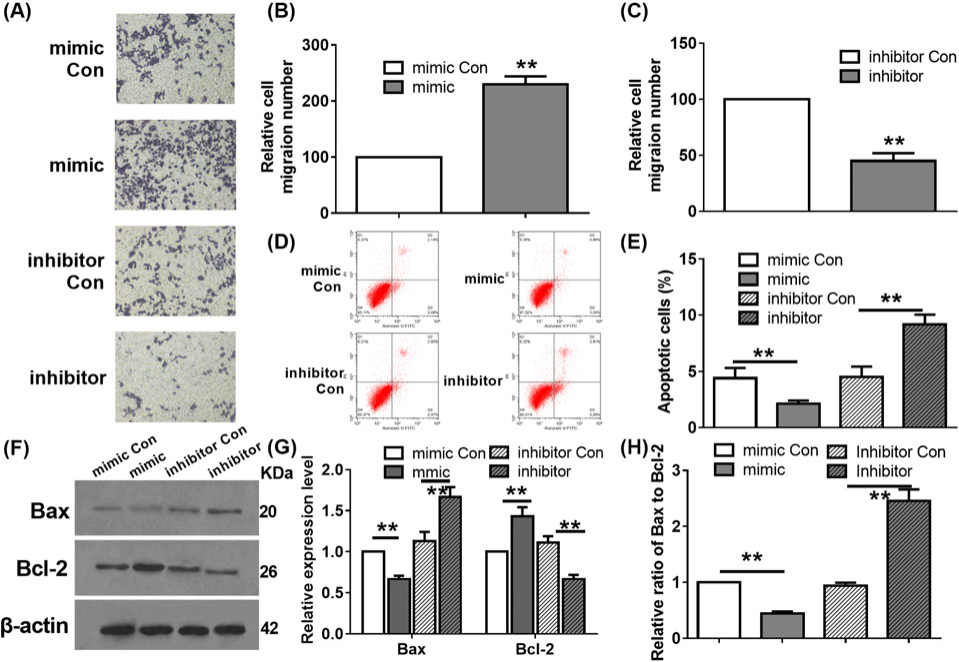
Effect of *miR-629-5p* on migration and apoptosis of LoVo cells (**A**) Effect of mimic (**B**) or inhibitor (**C**) transfection on cell migration was analyzed by Transwell assay. **P<0.01, Student’s *t* test. Data were obtained from three biological replicates. (**D**,**E**) Effects of mimic or inhibitor transfection on cell apoptosis were analyzed by Annexin V/PI analysis. **P<0.01, one-way ANOVA test. Data were obtained from three biological replicates. (**F**) Effect of mimic or inhibitor transfection on the expression levels of Bax and Bcl-2 was analyzed by WB assay. β-actin was used as the internal control. WB assay was performed in triplicate. (**G**) Effect of mimic or inhibitor transfection on the relative expression level of Bax and Bcl-2. **P<0.01, one-way ANOVA test. Data were obtained from three biological replicates. (**H**) Effect of mimic or inhibitor transfection on the relative ratio of Bax to Bcl-2. **P<0.01, one-way ANOVA test. Data were obtained from three biological replicates. WB: Western blot.

For assessing the potential functions of *miR-629-5p* in colorectal cancer cell line, *miR-629-5p* mimic, *miR-629-5p* inhibitor or relative negative controls were transfected into LoVo cells. Compared with mimic control (mimic Con), transfection with *miR-629-5p* mimic significantly improved its mRNA level, indicating the successful overexpression of *miR-629-5p* (Figure 2A). As shown in Figure 2B, overexpression of *miR-629-5p* led to an obviously enhanced cell viability compared with the mimic Con transfected cells. Transfection of *miR-629-5p* inhibitor significantly inhibited the mRNA level of *miR-629-5p* (Figure 2C), leading to a repressed cell viability than the inhibitor Con group (Figure 2D). Cell migratory activity of LoVo cells was also significantly enhanced in the *miR-629-5p* mimic group (Figure 3A,B). In contrast, transfection of *miR-629-5p* inhibitor resulted in an obviously decreased cell migratory activity (Figure 3A,C). These results indicate that *miR-629-5p* promotes cell proliferation and migration in LoVo cells.

### Effect of *miR-629-5p* on cell apoptosis in LoVo cells

To explore the effect of *miR-629-5p* on cell apoptosis, apoptotic rate of the transfected cells was determined by flow cytometry. As shown in Figure 3D,E, *miR-629-5p* overexpression resulted in a significantly decreased apoptosis level. In contrast, transfection of *miR-629-5p* inhibitor improved cell apoptosis compared with the *miR-629-5p* inhibitor Con transfected cells (Figure 3D,E). Further, the expression levels of apoptosis related Bcl-2 and Bax proteins were determined by WB assay. As shown in Figure 3F, *miR-629-5p* overexpression decreased the protein level of Bax and improved the protein level of Bcl-2 (Figure 3G), leading to a decreased ratio of Bax to Bcl-2 (Figure 3H). In contrast, transfection of *miR-629-5p* inhibitor showed contrary effects on the protein levels of Bcl-2 and Bax. These results suggest that *miR-629-5p* represses cell apoptosis in LoVo cells.

### *MiR-629-5p* directly targetting CXXC4 in LoVo cells

**Figure 4:**
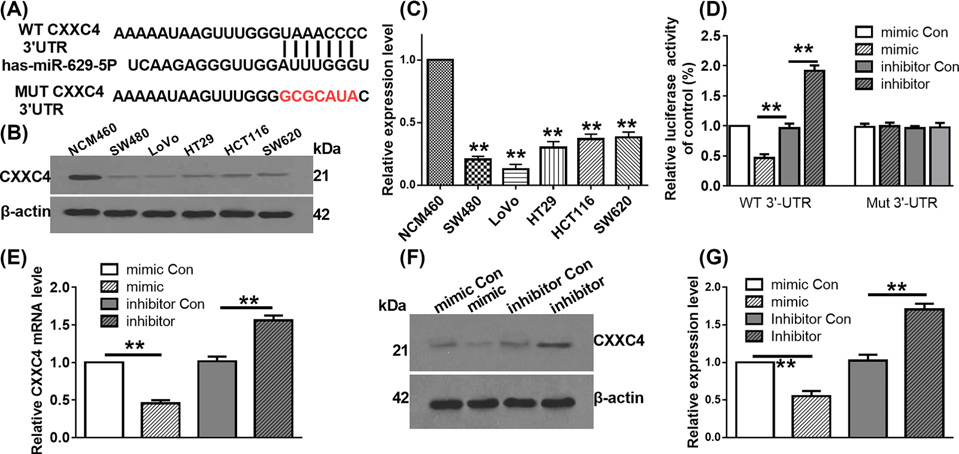
*miR-629-5p* directly regulating CXXC4 expression (**A**) Potential binding site between *miR-692-5p* and the 3′UTR of CXXC4 predicted by TargetScan 7.1 (http://www.targetscan.org/vert_71/). (**B**,**C**) Relative expression level of CXXC4 in colorectal cancer cell lines was determined by WB assay. β-actin was used as the internal control. **P<0.01, one-way ANOVA test. Data were obtained from three biological replicates. (**D**) Potential binding between *miR-629-5p* and the 3′UTR of CXXC4 was determined by dual-luciferase reporter assay. **P<0.01, one-way ANOVA test. Data were obtained from three biological replicates. (**E**) Effect of *miR-692-5p* mimic and *miR-692-5p* inhibitor on the mRNA level of CXXC4 was determined by qRT-PCR. GADPH was used as internal control. **P<0.01, one-way ANOVA test. Data were obtained from three biological replicates. (**F**,**G**) Effect of *miR-692-5p* mimic and *miR-692-5p* inhibitor on the protein level of CXXC4 was determined by WB assay. β-actin was used as internal control. **P<0.01, one-way ANOVA test. Data were obtained from three biological replicates.

Online bioinformatics analysis by TargetScan 7.1 version showed that CXXC4 was a potential target gene of *miR-629-5p* (Figure 4A). Expressions of CXXC4 in colorectal cancer cell lines were also analyzed by WB assay. As shown in Figure 4B,C, the protein level of CXXC4 in the tested colorectal cancer cell lines were all down-regulated, indicating the aberrant expression of CXXC4 in colorectal cancer cell lines. The direct regulation relationship between *miR-629-5p* and CXXC4 was further verified by dual-luciferase reporter assay. Co-transfection of *miR-629-5p* mimic or inhibitor with the luciferase reporter vector containing the wild-type 3′UTR of CXXC4 obviously repressed or improved luciferase activity, respectively (Figure 4D). However, *miR-629-5p* mimic or inhibitor showed no impact on the luciferase activities of the cells transfected with the mutant 3′UTR of CXXC4 (Figure 4D). Further analysis presented that the *miR-629-5p* mimic or inhibitor showed significantly negative or positive effect on the mRNA (Figure 4E) and protein levels of (Figure 4F,G) CXXC4 in LoVo cells, respectively. These results reveal that *miR-629-5p* directly binds to the 3′UTR of CXXC4 and negatively regulated its expression at post-transcriptional level.

### The role of CXXC4 in the oncogenic function of *miR-625-5p* in LoVo cells

**Figure 5:**
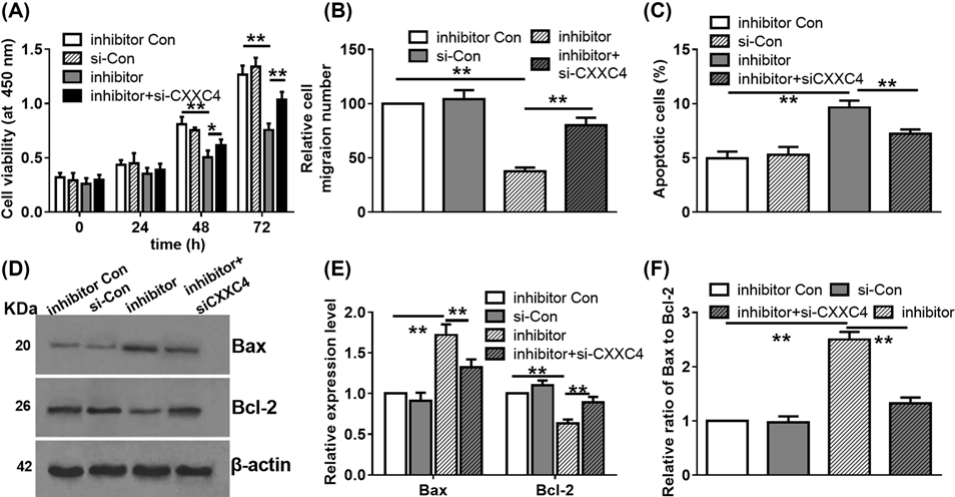
CXXC4 involves in the oncogenic effects of *miR-629-5p* on colorectal cancer progression (**A**) Effect of co-transfection of siCXXC4 and *miR-629-5p* inhibitor on cell viability was determined by CCK-8 assay. *P<0.05, **P<0.01, one-way ANOVA test. Data were obtained from three biological replicates. (**B**) Effect of co-transfection of siCXXC4 and *miR-629-5p* inhibitor on cell migration was determined by Transwell assay. **P<0.01, one-way ANOVA test. Data were obtained from three biological replicates. (**C**) Effect of co-transfection of siCXXC4 and *miR-629-5p* inhibitor on cell apoptosis was determined by Annexin V/PI analysis. **P<0.01, one-way ANOVA test. Data were obtained from three biological replicates. (**D**) Effect of co-transfection of siCXXC4 and *miR-629-5p* inhibitor on the expression levels of Bax and Bcl-2 was analyzed by WB assay. β-actin was used as the internal control. WB assay was performed in triplicate. (**E**) Effect of co-transfection of siCXXC4 and *miR-629-5p* inhibitor on the relative expression levelS of Bax and Bcl-2. **P<0.01, one-way ANOVA test. Data were obtained from three biological replicates. (**F**) Effect of co-transfection of siCXXC4 and *miR-629-5p* inhibitor on the relative ratio of Bax to Bcl-2. **P<0.01, one-way ANOVA test. Data were obtained from three biological replicates.

To illustrate whether CXXC4 involves in the oncogenic function of *miR-625-5p*, si-CXXC4 was transfected with *miR-625-5p* inhibitor in LoVo cells to knockdown CXXC4 level. As shown in Figure 5A, the cell viabilities of different groups were similar at 24 h. After that, transfecting *miR-629-5p* inhibitor showed obvious inhibitory effect on cell viability and CXXC4 knockdown exhibited the ability to rescue this repressed cell viability. In addition, CXXC4 knockdown also partly recovered the decreased cell migratory ability induced by the decreased *miR-625-5p* level (Figure 5B). Besides, the enhanced cell apoptosis (Figure 5C), the aberrant expressions of Bcl-2 and Bax proteins (Figure 5D,E), and the enhanced ratio of Bax to Bcl-2 (Figure 5F) in the *miR-625-5p* inhibitor transfected cells were also partly abrogated by CXXC4 knockdown. These results reveal that miR-625-5p is a tumor promoter via targetting CXXC4 in LoVo cells.

### Effect of *miR-629-5p*/CXXC4 axis on the 5-fluorouracil sensitivity of LoVo cells

**Figure 6:**
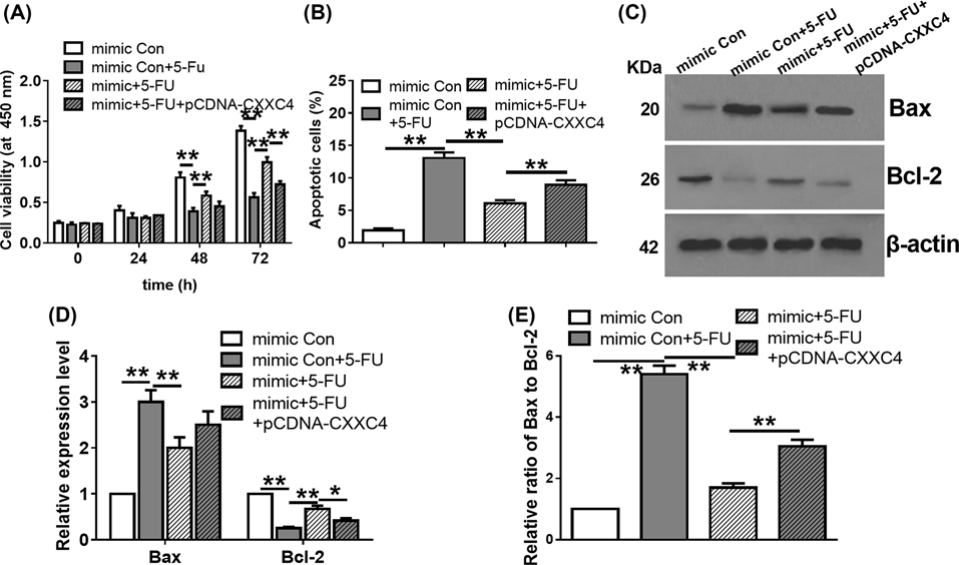
*miR-629-5p* contributes sensitivity to 5-fluorouracil in LoVo cells through CXXC4 (**A**) Effect of coexpression of *miR-629-5p* and CXXC4 on cell sensitivity to 5-FU (0.6 μg/ml). Cell viability was determined by the CCK-8 assay. **P<0.01, one-way ANOVA test. Data were obtained from three biological replicates. (**B**) Effect of coexpression of *miR-629-5p* and CXXC4 on cell apoptosis under the treatment of 5-FU was analyzed by Annexin V/PI analysis. **P<0.01, one-way ANOVA test. Data were obtained from three biological replicates. (**C**) Effect of coexpression of *miR-629-5p* and CXXC4 on the expression levels of Bax and Bcl-2 under the treatment of 5-FU was analyzed by WB assay. β-actin was used as the internal control. WB assay was performed in triplicate. (**D**) Effect of coexpression of *miR-629-5p* and CXXC4 on the relative expression level of Bax and Bcl-2 under the treatment of 5-FU. * *P*<0.05, **P<0.01, one-way ANOVA test. Data were obtained from three biological replicates. (**E**) Effect of coexpression of *miR-629-5p* and CXXC4 on the relative ratio of Bax to Bcl-2 under the treatment of 5-FU. **P<0.01, one-way ANOVA test. Data were obtained from three biological replicates.

We also explored the role of *miR-629-5p*/CXXC4 axis on the 5-fluorouracil sensitivity of LoVo cells. As shown in Figure 6A, the cell viabilities of different groups were similar at 24 h. After that, transfection of *miR-629-5p* mimic decreased the inhibitory effect of 5-FU on cell viability. Further, coexpression of CXXC4 in mimic/5-FU cell repressed cell viability at 72 h. Overexpression of *miR-629-5p* restored the 5-FU induced apoptosis of LoVo cells compared with the mimic Con/5-FU group (Figure 6B). We also examined the expression level of apoptotic marker proteins (Bax and Bcl-2) by WB assay (Figure 6C). Transfection of *miR-629-5p* mimic decreased the expression level of Bax (Figure 6D), increased the expression level of Bcl-2 (Figure 6D), and decreased the ratio of Bax to Bcl-2 than the mimic Con/5-FU group (Figure 6E). Notably, the changes in apoptosis, the expression levels of Bax and Bcl-2, and the ratio of Bax to Bcl-2 were attenuated by coexpression of CXXC4 in the mimic/5-FU group (Figure 6B–E). These data suggest that the *miR-629-5p* contributes to 5-FU sensitivity partly via regulating CXXC4 expression in LoVo cells.

## Discussion

Emerging evidence has revealed the varied functions of miRNAs in colorectal cancer progression. For example, *miR-19a* contributed to colorectal cancer proliferation and migration by targetting TIA1 [20]*. MiR-145* inhibited human colorectal cancer cell migration and invasion through PAK4-dependent pathway [21]. *MiR-429* promoted tumor growth and metastasis in colorectal cancer by directly targetting HOXA5 [22]. *MiR-542-3p* functioned as a tumor repressor in colorectal cancer by targetting OTUB1 [23]. However, the role of *miR-629-5p* in colorectal cancer remains unclear. Here, we found that *miR-629-5p* was up-regulated both in colorectal cancer tissues and cell lines. However, its extent of up-regulation in colorectal cancer is significantly lower than that in other cancer types [12,24], indicating a potential varied regulation of *miR-629-5p* in different types of cancer. Additionally, there was no significant correlation between the metastasis of tested cell lines and the expression level of *miR-629-5p*. Similar to previous studies [12,13,24], *miR-629-5p* functioned as an oncogene by promoting proliferation and migration and repressing apoptosis and drug sensitivity. These results suggest that *miR-629-5p* is a potential biomarker and therapy target for colorectal cancer.

Previous studies have showed that *miR-629-5p* involves in tumor progression via down-regulating FOXO3 [12], Testis-specific Y-like protein 5 [24], or TRIM33 [11] in different types of cancer. In the present study, we found that CXXC4 was down-regulated in the tested colorectal cancer cell lines, which is similar to a recent report [19]. Further analyses confirmed that CXXC4 was directly down-regulated by *miR-629-5p* and involved in the oncogenicity of *miR-629-5p*. In contrast, regulating CXXC4 expression level showed an earlier obvious rescue effect on cell viability than on drug sensitivity, indicating a potential and more important role of CXXC4 on cell viability.

CXXC4 has been identified as a tumor suppressor and was down-regulated in renal cell [14], gastric [15,16], and colorectal cancers [19]. Its mRNA level is also associated with clinicopathological parameters and patient survival in myelodysplastic syndrome [25]. As a transcription factor, CXXC4 functions via multiple targets in various cancer types. In gastric cancer, CXXC4 abrogated the interaction of ERK1/2 with MEK1/2 via binding to ERK1/2 to inactivate MAPK signaling [15]. It also showed the ability to interact with disheveled to disrupt the association of disheveled with Axin-GSK-3b and consequently activate GSK-3b to promote the phosphorylation and degradation of β-catenin, inhibiting Wnt signaling [16]. Besides, CXXC4 may contribute to the activation of caspase 3 [26]. Recent report indicated that CXXC4 activated GDF15 transcription by improving the interaction of Sp1 with GDF15 promoter to strengthen cell apoptosis [17]. In renal cell carcinoma, down-regulation of CXXC4 by siRNA resulted in the up-regulation of some cellular proliferation related genes (*FGF18, EGR1*, and *MYCN*), the down-regulation of apoptosis inhibitor proteins BIRC7 and XAF1, and some other important tumor progression factors [14]. Therefore, *miR-629-5p* can form a complex regulating network via down-regulating CXXC4.

As a tumor repressor, the regulation of CXXC4 expression in transcription level has been reported. In colorectal cancer, the B cell-specific Molony murine leukemia virus integration site1, a component of the polycomb repressive complex, can bind to the promoter region of CXXC4 and then repress its transcription [19]. In gastric cancer, the enhancer of zeste homolog 2 was enriched on CXXC4 promoter region and down-regulated its expression [16]. However, whether CXXC4 is regulated at post-transcriptional level remains unclear. Our result is the first report, which provides the evidence that CXXC4 is also directly down-regulated by miRNA via binding to its 3′UTR. Notably, regulating the CXXC4 level by overexpression or RNAi did not completely rescue the *miR-629-5p* induced changes in cell properties. This phenomenon can be explained by the complex regulatory mechanism of both and CXXC4.

In summary, our data reveal that the expression level of *miR-629-5p* is enhanced in colorectal cancer tissues and cell lines. *MiR-629-5p* functions as a tumor promoter by improving proliferation and migration and repressing apoptosis and 5-FU sensitivity in colorectal cancer progression by directly down-regulating CXXC4. Besides, this research is also the first study provides evidence that CXXC4 is regulated by miRNA at post-transcriptional level.

## Abbreviations

CXXC4: CXXC finger protein 4
5-FU: 5-Fluorouracil
qRT-PCR: quantitative real-time PCR
